# Neuronal substrates underlying stress resilience and susceptibility in rats

**DOI:** 10.1371/journal.pone.0179434

**Published:** 2017-06-16

**Authors:** Fabia Febbraro, Katrine Svenningsen, Thao Phuong Tran, Ove Wiborg

**Affiliations:** 1 Danish Research Institute of Translational Neuroscience (DANDRITE) Aarhus University, Aarhus C, Denmark; 2 Focused Research Unit for Molecular Diagnostic and Clinical Research IRS-Center Sonderjylland, Laboratory Center, Hospital of Southern Jutland, Åbenrå, Denmark; 3 Translational Neuropsychiatry Unit, Department of Clinical Medicine, Aarhus University, Risskov, Denmark; 4 Department of Clinical Medicine, Health, Aarhus University, Aarhus C, Denmark; Technion Israel Institute of Technology, ISRAEL

## Abstract

**Background:**

Stress and stressful life events have repeatedly been shown as causally related to depression. The Chronic Mild Stress rat model is a valid model of stress-induced depression. Like humans, rats display great heterogeneity in their response to stress and adversity. Hence some individuals are stress-sensitive and prone to develop depression-like behaviour in response to modest stressors, while others are stress-resilient and remain essentially symptom free.

**Objectives:**

Compared to the large body of research, which describes stress-induced maladaptive neurobiological changes, relatively little attention has been devoted to understand resiliency to stress. The aim of the present study was to identify changes in neuronal activity, associated with stress-resilient and stress-susceptible chronic mild stress endophenotypes, by examining *c-Fos* expression in 13 different brain areas. Changes in *c-Fos* expression have been reported as associated to stressful conditions.

**Methods:**

Stress-induced modulation of neuronal activation patterns in response to the chronic mild stress paradigm was mapped using the immediate early gene expression *c-Fos* as a marker. Quantification of the *c-Fos*-like immunoreactivity responses was done by semi-automated profile counting procedures and design-based stereology.

**Results:**

Exposure to chronic mild stress significantly altered *c-Fos* expression in a total of 6 out of 13 investigated areas. Chronic mild stress was found to suppress the *c-Fos* response within the magnocellular ventral lateral geniculate nucleus of both stress subgroups. In the the lateral and ventral orbital cortices of stress-resilient rats, the *c-Fos* like immunoreactivity response was also repressed by stress exposure. On the contrary the *c-Fos* response within the amygdala, medial habenula, and infralimbic cortex was increased selectively for the stress-susceptible rats.

**Conclusions:**

The study was initiated to characterize neuronal substrates associated with stress-coping mechanisms. Six areas, all of which represents limbic structures, were found to be sensitive to stress exposure. The effects within these areas associate to the hedonic status of the rats. Hence, these areas might be associated to stress-coping mechanisms underlying the chronic mild stress induced segregation into stress-susceptible and stress-resilient endophenotypes.

## Introduction

Coping strategies are essential to minimize the impact of stress and determine the extent of resilience or susceptibility of each individual [1, 2]. How individuals cope when exposed to acute and chronic stressors plays a central role for the potential development of stress-related disorders. The use of passive coping is often a characteristic of Major Depressive Disorder (MDD), manifested by behaviors such as social withdrawal and agitation [3]. Coping styles varies among individuals and according to the severity of specific stressful situations. At the physiological level the neuroendocrine system is activated in response to stress, and while some individuals are affected by stressful events others are resilient and respond to stress by applying adaptive mechanisms to maintain their *status quo* both at behavioural and physiological level [4–6]. The biological basis of stress response and coping strategies is not clearly defined, and its understanding is essential for a better comprehension of the aetiology of stress-related disorders such as MDD [7–9].

Stress is an essential physiological response that is highly conserved during evolution [10], and like humans, animals also use coping strategies when exposed to stressors [11]. Rodents can express both active and passive coping strategies such as defensive or aggressive behaviors contra immobility, submission and helpless behavior [12–15]. These behaviors, which reflect stress responses, can be measured and used as read-outs on models of stress and coping in humans.

The chronic mild stress (CMS) model is an extensively validated model of stress-induced anhedonia, which is a core symptom of MDD [13, 16]. Anhedonia is characterized by diminished interest or pleasure in response to stimuli that were perceived as rewarding during the premorbid state.

The hedonic state of CMS animals is quantified by the sucrose consumption test (SCT). It has previously been shown that some rats are resilient and can cope with the heterotypic stress regime in order to maintain homeostasis, while some are much more vulnerable or susceptible and enter an anhedonic-like state including reduced reward sensitivity [16]. The resilient and susceptible endophenotypes of the CMS model makes it possible not only to study general effects of stress, but also provide an opportunity to study the biological basis of differential stress-coping mechanisms.

In the present study we investigate stress coping mechanisms by examining several brain regions to identify areas activated during stress. Intrinsic differences in the anatomical and functional connectivity of the brain may account for why some individuals are more vulnerable to stress than others. Therefore we wanted to investigate neuronal population and circuitries that are responsive to stress exposure in general, but also neuronal populations that are distinctly activated in susceptible and resilient CMS endophenotypes, respectively.

Analysis of *c-Fos* immunoreactivity (c-Fos-ir) has proved to be a useful tool for mapping cellular and functional pathways involved in emotional processes and neuroendocrine responses to a variety of acute [17–20] and heterotypic chronic stressors [21–23] (for reviews on *c-Fos* technology see [24, 25]). *c-Fos* belongs to the family of Immediate Early Genes [26] which represents a permanent and lasting response mechanism facilitating stimulus-transcription-coupling in the first round of response to stimuli [27].

Assuming that *c-Fos* expression is responsive to stress exposure [28], we hypothesized I) that stress susceptible rats as compared with unchallenged control rats would display differential c-Fos-ir profiles revealing neuronal populations affected by chronic stress; II) that stress susceptible rats as compared with stress resilient rats would also display differential c-Fos-ir profiles; and III) that such neuronal populations might be associated to stress-coping mechanisms underlying the CMS induced segregation into stress-susceptible and stress-resilient endophenotypes.

Stress resilience may be mediated by adaptive changes in several neural circuits that regulate reward, fear, emotional reactivity and social behavior, which are altogether believed to mediate successful stress coping [29]. Therefore these investigations should in a translational perspective increase our understanding of the pathogenesis and pathophysiology of stress and MDD.

## Methods

### Subjects

Outbred male Wistar rats (Taconic M&B, Ry, Denmark) at age 5–6 weeks, weighing 100–120 g, were used in the study. The animals were singly housed, food and water was available *ad libitum*, and animals were kept on a standard 12-h light/dark cycle except when one of these parameters was changed because of the stress regime. All animal procedures were approved by the Danish National Committee for Ethics in Animal Experimentation (2008/561–447).

### Sucrose consumption test

During the first five weeks, the animals were trained to consume a palatable sucrose solution (1.5%) to quantify the hedonic state. The sucrose test was conducted twice a week during the first 2 weeks and once a week during the following 3 weeks. During the stress period, the sucrose consumption test was performed once a week. Animals were food and water deprived 14 h prior to the test, which was a 1-hour period of free access to a bottle with sucrose solution. Rats with a mean sucrose intake below 8.5 g were designated as innate low-drinkers and excluded from the study. Baseline sucrose consumption was defined as the mean sucrose consumption during the final two sucrose tests conducted before initiation of the stress paradigm. Rats were assigned to the experimental groups of stress exposed and naïve animals, in order to have equal mean and S.D. values, based on their baseline sucrose intake.

### Chronic mild stress

According to individual baseline sucrose intake, animals were divided into two groups and placed in separate rooms. One group, the CMS group, was exposed to 4 weeks of chronic mild stressors and the other, the control group, was left unchallenged. Following the final sucrose test Friday morning, stress exposure was continued, and rats were euthanized Monday morning. The unchallenged control group was food and water deprived 14 h before sucrose consumption test only.

**Fig 1:**
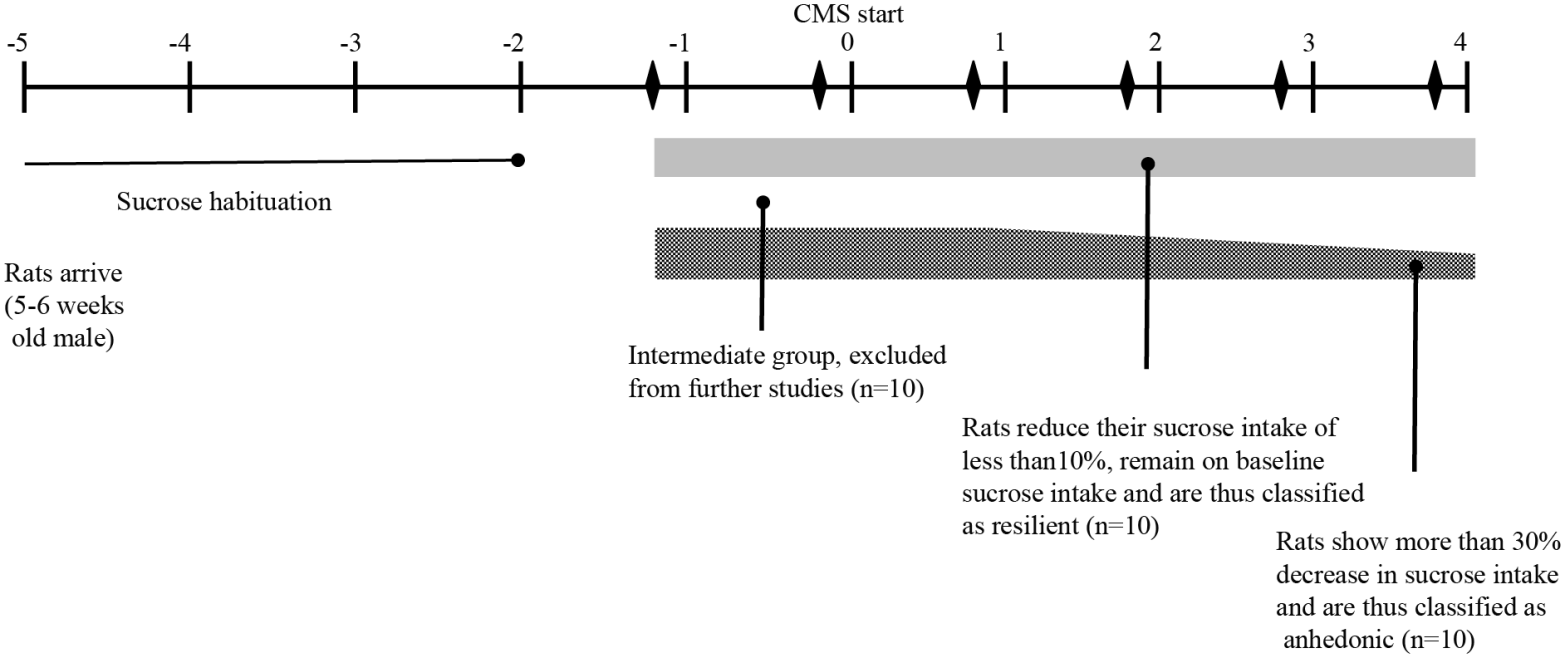
Study design showing the experimental outline and time course. (modified from [70]).

The stress procedure was optimized in our laboratory and performed as described in [30]. Briefly the stress protocol consisted of seven different stress conditions each lasting 10 to 14 h. One period of intermittent illumination, stroboscopic light (2 Hz), grouping, food or water deprivation, and two periods of soiled cage (400 mL) and no stress, and three periods of 45° cage-tilting. Following exposure to stress, rats were characterized as anhedonic-like (defined as a >30% within-subject decrease in sucrose intake) or resilient (defined as a < 10% within subject decrease in sucrose intake). Rats not corresponding to either criterion were excluded from the experiment. For the purposes of the present study 10 control, 10 resilient, and 10 anhedonic-like (stress susceptible) rats were included (Fig 1).

### Tissue processing

After four weeks of stress regime, the animals were deeply anesthetized with an intraperitoneal overdose of sodium pentobarbital (60 mg/ml, 100 mg/kg) and perfused through the ascending aorta with 100 ml 0.9% saline, followed by 200 ml of ice cold 4% paraformaldehyde (pH 7.2–7.4) in phosphate buffer. The brains were removed from the skull and post-fixed for 24 hours at 4°C in the same solution. The brains were transferred to a solution consisting of 25% sucrose (diluted in phosphate buffer, pH 7.0–7.4) supplemented with 10% sodium azide and stored at 4°C until further processing. The cerebellum was dissected from the brains in order to facilitate consistent mounting (Tissue-Tek O.C.T. Compound, Sakura Finetek). Coronal sections (40 μm) were sliced using a cryostat (CM3050 S, Leica Microsystems). Sections from respectively the forebrain and midbrain were collected in two separate series of every 10 slices and stored in cryoprotectant antifreeze solution (25% ethylene glycol and 25% glycerin in a 0.05 M phosphate-buffered saline (PBS)) at –20°C until further processing. The forebrain region (4.70 to -1.40 mm relative to bregma according to rat brain atlas of Paxinos & Watson 1998 [31]) and the midbrain region (-2.12 to -6.30 mm relative to bregma) were collected.

### Immunohistochemistry

Immunohistochemical staining was carried out on free-floating sections using c-Fos polyclonal antibody (raised against an epitope, mapping a 15 amino acid sequence of the N-terminus of human c-FOS protein, sc-52, 1:500, Santa Cruz Biotechnology). The sections were rinsed three times in potassium-phosphate buffer (KPBS) between each incubation period as well as before and after quenching procedures. All procedures were done at room temperature and during gentle rotation. Each series, from respectively the forebrain or midbrain regions, were processed simultaneously across all three groups in order to avoid batch effects. Control sections without primary antibody were included. The sections were quenched for 10 min in 3% H_2_O_2_ and 10% methanol in KPBS. Blocking of unspecific binding sites with 5% appropriate serum was followed by overnight incubation at room temperature with primary antibody in 2.5% serum. Additional blocking (10 min) with 1% serum was done before incubating with biotinylated anti-rabbit-IgG secondary antibody (BA-1100 Vector Laboratories, 1:200) in 1% normal serum. An avidin–biotin–peroxidase procedure (ABC Elite, Vector Laboratories) with 3,3-diaminobenzidine (DAB) as the chromogen was used to visualize c-Fos-immunoreactive (ir) cells. Sections from each series were mounted onto glass slides prepared with chrome-alum-coating in chronological order from anterior to posterior. The glass slides were then left to dry at room temperature for 48 hours, then dehydrated in ascending alcohol concentrations, cleared in xylene and cover slipped using DPX Mountant for histology (Sigma). The staining procedure generally yielded low background staining and differential staining intensities of c-Fos-ir cells.

### Stereology by the optical fractionator method

Unilateral estimation of c-Fos-ir cell numbers was calculated according to the optical fractionator formula [32, 33]. For the stereological examination a systematic random sampling of every fifth coronal section throughout the entire habenular complexes was analyzed. This provides 9–10 sections in a series. Sampling was performed using the NewCast Module in VIS software (Visiopharm A/S, Horsholm, Denmark) and an eclipse Ni (Nicon) microscope applied with an Olympus DP73 digital camera (17.28 megapixel resolution). Furthermore, the stage of the microscope was equipped with an x-y motorized stage and high-precision linear encoder. A low-power (4x, 0.13 plan Fluor) objective was used to delineate the borders of the area. Counting was performed with a 20x (0.75 plan Apo) objective, using c-Fos-ir nuclei as the counting unit in all regions. The counting frame was placed randomly on the first counting area and systematically moved through all counting areas until the entire delineated region was sampled. c-Fos-ir neurons were counted when the nucleus came into focus inside the optical dissector without touching the exclusion lines of the counting frame. The area of the counting frame was 2025 μm^2^ (45x45 μm) and a quadratic step length of 75 μm was applied. The counting frame was placed randomly on the first counting area and systematically moved through all regions of interest until the entire delineated region was sampled. Average tissue shrinkage in the z-axis (60x oil, 1.40 plan Apo) or c-Fos-ir cell morphology (20x, 0.75 plan Apo, using the “measure diameter” module of the VIS software) did not differ across groups. The estimates of the total numbers of neurons were calculated according to the optical fractionator formula, and a coefficient of error < 0.10 was accepted [32, 33]. Each immunoreactive cell was classified as being light, medium or dark, according to respective levels of c-Fos-ir. The cells were scored according to a predefined colour intensity threshold corrected for background staining.

### Densitometric procedures

Digital microphotographs covering an area of 700 x 525 μm2 were used for densitometric quantification (eclipse Nicon microscope, DP73 Olympus digital camera, and 10x (0.25 plan) objective) of 13 different substructures unilaterally at 3 rostrocaudal levels, within each structure, throughout different brain regions. The microphotographs were analyzed for c-Fos-ir density, and staining intensity.

The digital microphotographs were processed using the ImageJ software package (version 1.48, National Institute of Health, USA). The software was calibrated (Kodak No. 3 Calibrated Step Tablet) to an optical density (OD) range of 0.05 to 3.05. Contrast settings and background subtraction of the microphotographs were optimized. An outline of particles or c-Fos-ir profiles, above a defined size-threshold of 12μm^2^ and with a circularity factor of 0.01–1.00 was generated. When present, artefacts were manually removed. After sufficient refinement, the final outline was superimposed onto a 16-bit version of the original picture to extract OD and area of each c-Fos-ir profile as well as the density of c-Fos-ir profiles. OD readings were corrected for background staining (S1 Fig).

Numerous pilot studies had been conducted to determine the optimal setting for the densitometric procedures. However, c-Fos-ir profiles corresponding to c-Fos-ir cells classified as light during the stereological procedures were lost as background noise in the densitometric analyses. Hence the profiles analyzed with the densitometric methods correspond to the c-Fos-ir cells classified as medium and dark intensity cells during the stereological procedures.

### Statistics

Gaussian distribution was assumed from analysis by D’Agostino-Pearson omnibus normality test. Potential outliers were evaluated by Grubbs’ (alpha = 0.01) method. A one-way ANOVA was used to analyse differences among groups followed, when appropriate, by post hoc Tukey´s multiple comparison test. For the sucrose consumption in different time points, we used two-way repeated measure ANOVA followed when appropriate by post hoc analysis. Statistical significance was set at p < 0.05. All results are presented as mean ± SEM. GraphPad Prism 6 was used for the statistical analyses.

## Results

### The CMS regime—Sucrose consumption

**Fig 2:**
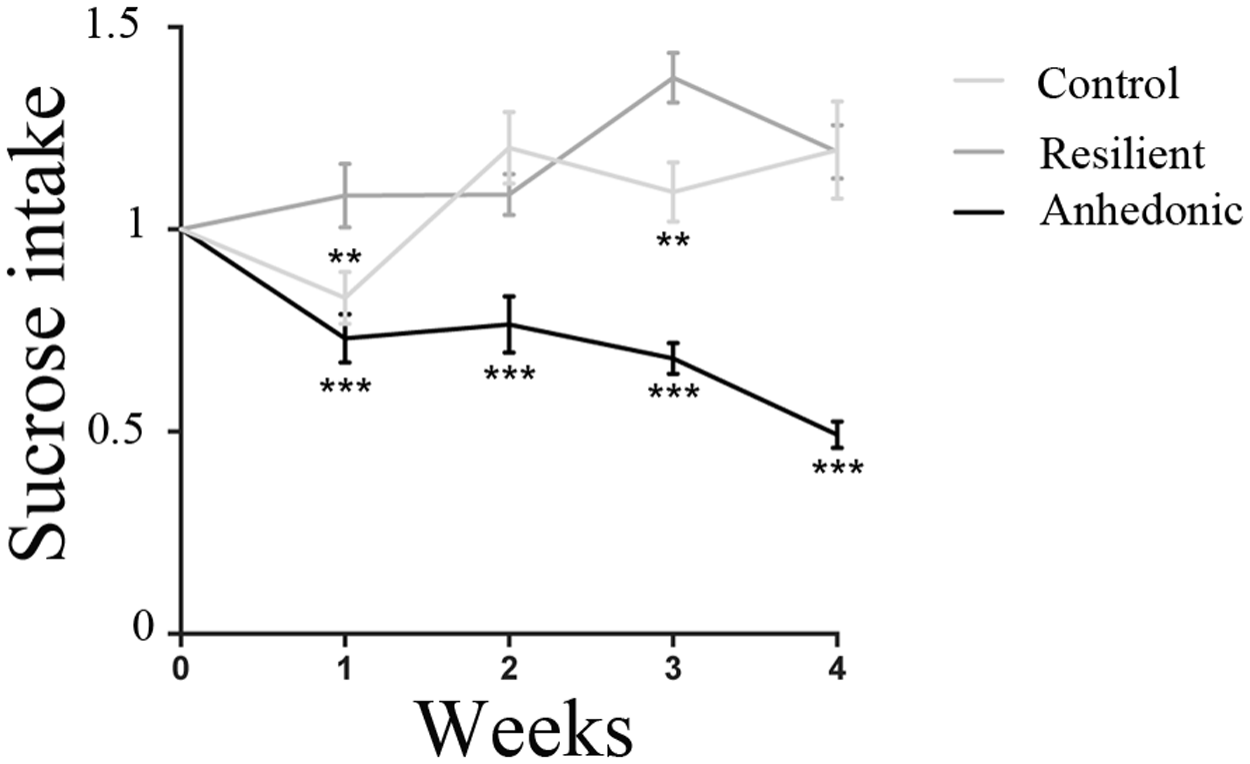
Sucrose consumption following four weeks of the CMS regime. Four weeks exposure to chronic mild stressors resulted in a significant decrease of sucrose consumption of the anhedonic animals (n = 10) when compared to unchallenged controls (n = 10). The graph shows sucrose consumption of the control (n = 10), resilient (n = 10) and anhedonic-like (n = 10) animals at 4 different time points (week 1; week 2; week 3 and week 4). Significant differences on sucrose consumptions were detected among goups (p<0,0001) at different time points (p = 0,0084). The sucrose intake of anhedonic-like animals was significantly diminished when compared to control and resilient animals. Two-way ANOVA followed by post hoc Tukey´s multiple comparison test. * p<0,05; **p<0,01; ***p<0,001. Data is presented as mean (±SEM) sucrose intake, indexed to baseline values.

The effect of CMS on the hedonic response was assessed by weekly sucrose consumption tests of respectively control and CMS animals. The sucrose consumption from CMS animals was used to monitor the graduation of the stress-induced segregation into anhedonic-like, stress-resilient and intermediate phenotypes. Following four weeks of the CMS regime, the presence of a general stress-induced hedonic state of all CMS rats was observed when comparing their sucrose intake to that of the unchallenged control group (p < 0.0001; Fig 2). Furthermore, among the animals exposed to stress, rats showing more than 30% decrease in sucrose intake, indicating a stress-induced decrease in sensitivity to reward, were defined as anhedonic-like. On the contrary, stressed rats decreasing their sucrose intake by less than 10% were designated resilient. From both subgroups 10 rats were randomly selected for the present study. Intermediate rats were excluded from the experiment. The unchallenged control rats remained on a stable level of sucrose intake. Fig 2 shows the mean sucrose intake of these three groups each week, during 4 weeks of the CMS protocol. Significant differences on sucrose consumptions were detected among groups (F (2,27) = 39,72 p<0,0001) at different time points (F (4,108) = 3,608; p = 0,0084). At week one there was significant difference in sucrose consumption in resilient animals *versus* control (p = 0,0148) and anhedonic (p = 0,0003); in week 2 and 4 sucrose consumption was comparable in control and resilient animals (p = 0,4950 in week 2 and p = 0,9989 in week4), while the sucrose consumption in the anhedonic group was drastically reduced both when compared with the control group (34%; p<0,0001, in week 2 and 59%; p<0,0001 in week 4) and to the resilient group (36%; p = 0,0012 in week 2 and 66%; p<0,0001 in week 4). In week 3 the anhedonic group showed a significant reduction in sucrose consumption when compared to the control group (p<0,0001) and to the resilient group (p<0,0001). The resilient group was also different from the control at this particular time point (p = 0,0052). Two-way repeated measure ANOVA.

### Stereology—The medial and lateral habenula

**Fig 3:**
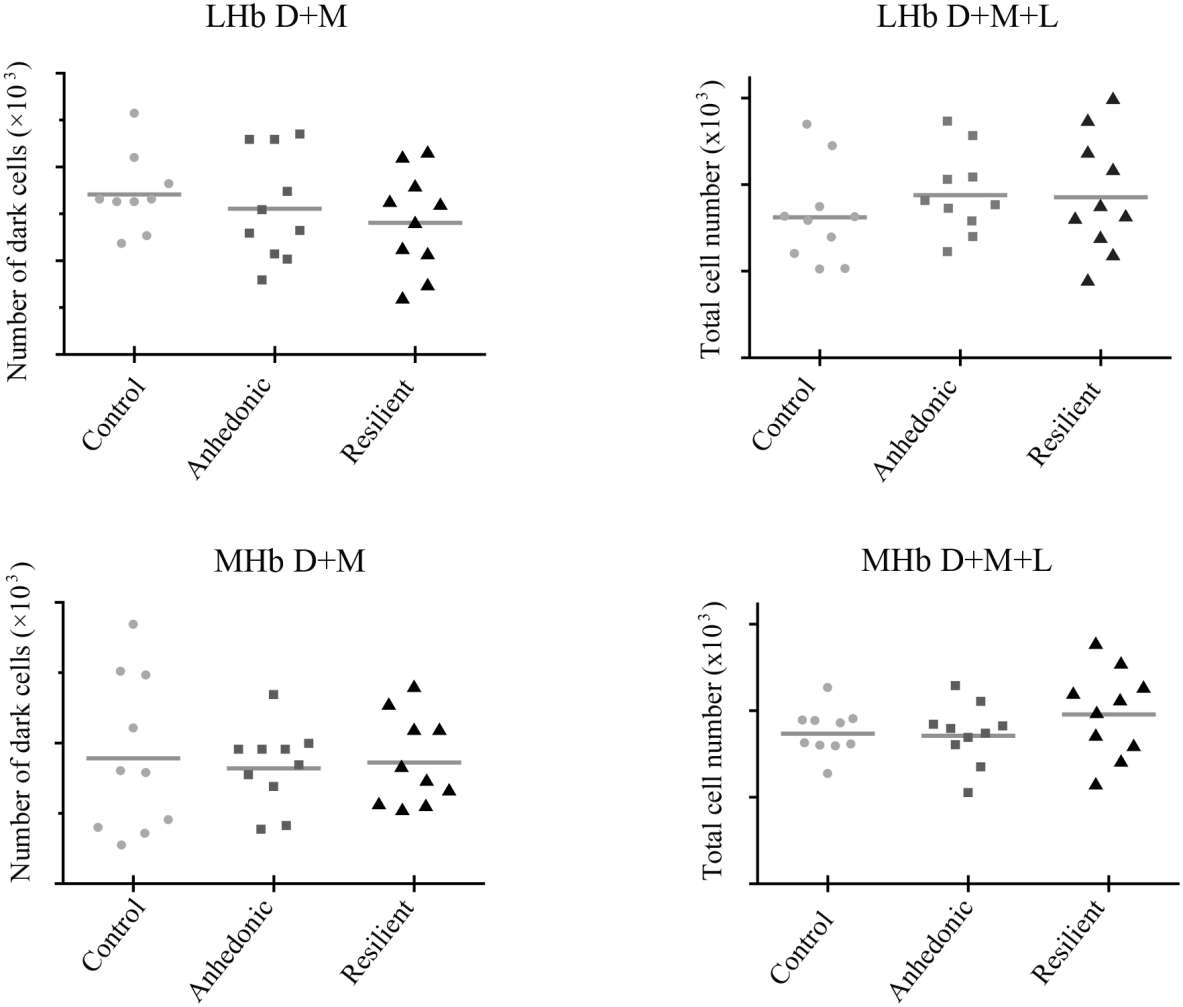
Stereological estimates of total c-Fos-ir in the habenular complexes. The estimates of total c-Fos-ir in the lateral (A) and medial (B) habenular complexes analysed with stereology. In plot A and B number of cells counted in the LHb as the sum of dark and medial c-Fos-ir and the sum of dark, medial and light c-Fos-ir, respectively. In plot C and D number of cells counted in the MHb as the sum of dark and medial c-Fos-ir and the sum of dark, medial and light c-Fos-ir, respectively. One-way ANOVA. LHb, lateral habenula; MHb, medial habenula.

Stereological quantification of total c-Fos-ir positive cell numbers within respectively the Medial habenula (MHb) and lateral habenula (LHb), did not show any difference among the three groups. For the MHb the total number of cells was 3.47 (*10^3) ± 0.17 in the control group; 3.45 (*10^3) ± 0.22 in the anhedonic-like group; and 3.91 (*10^3) ± 0.32 in the resilient group (F(2,27) = 1.216; p = 0,3121. For the LHb the total number of cells was 3.24 (*10^3) ± 0.35 in the control group; 3.76 (*10^3) ± 0.3 in the anhedonic group and 3.71 (*10^3) ± 0.43 in the resilient group (F(2,27) = 0.6034; p = 0.5541) (Fig 3A–3D, Table 1).

**Table 1:** Results summary.

**Stereology**		**Control**		**Anhedonic-like**		**Resilient**		**A vs C**		**R vs C**		**R vs A**	
**Brain area One-way ANOVA (F and p values)**	**parameter**	**mean**	**SEM**	**mean**	**SEM**	**mean**	**SEM**	**Post hoc test**	**%diff (↑↓)**	**Post hoc test**	**%diff (↑↓)**	**Post hoc test**	**%diff (↑↓)**
**MHb** F(2,27) = 1.216; p = 0,3121	total n. (*10^3)	3.47	0,17	3.45	0,22	3.91	0,32		-4.4		44.6		49.0
**LHb** F(2,27) = 0.6034; p = 0,5541	total n. (*10^3)	3.24	0,35	3.76	0,30	3.71	0,43		51.2		46.7		-4.5
**MHb** F(2,27) = 0.07684; p = 0,9262	D+M (*10^3)	0.90	0.18	0.82	0.09	0.86	0.10		-7,0	0,9859	-2,9		4,1
**LHb** F(2,26) = 0.8055; p = 0,4577	D+M (*10^3)	0.85	0.07	0.78	0.09	0.70	0.08		-7.5		-15		-7.5
**Densitometry**		**Control**		**Anhedonic-like**		**Resilient**		**A vs C**		**R vs C**		**R vs A**	
**Brain area (bregma levels) One-way ANOVA (F and p values)**	**parameter**	**mean**	**SEM**	**mean**	**SEM**	**mean**	**SEM**	**Post hoc test**	**%diff (↑↓)**	**Post hoc test**	**%diff (↑↓)**	**Post hoc test**	**%diff (↑↓)**
**BLA** (-2,12; -2,28; -2,44)													
F(2,26) = 0,3686; p = 0,6953	Density	19,8	2,4	21,0	2,1	22,3	1,5		6,1		12,4		6,0
F(2,26) = 3,779; p = 0,0363*	Int (*10^2)	13,9	1,1	**14,9**	**1,1**	**11,1**	**0,9**	0,7887	7,1	0,1526	-19,9	0,0347*	-25,2
F(2,26) = 0,7369; p = 0,4883	C.int.	16,6	2,5	17,8	2,4	14,3	1,4		7,4		-13,9		-19,9
**MHb** (-3.3;-3.46;-3,62)													
F(2,27) = 1,993; p = 0,1598	Density	11,6	1,2	9,1	1,0	9,1	0,8		-20,9		-21,4		-0,6
F(2,27) = 4,331; p = 0,0234*	Int (*10^2)	**5,4**	**0,3**	**7,1**	**0,4**	6,9	0,6	0,0327*	25,8	0,9607	5,1	0,0588	-16,5
F(2,26) = 1,106; p = 0,3460	C.int.	2,5	0,4	2,1	0,2	1,9	0,2		-14,4		-23,9		-11,1
**LHb** (-3,3; -3,46; -3,62)													
F(2,27) = 2,258; p = 0,1240	Density	9,2	1,6	7,2	0,9	5,8	0,6		-22,0		-36,4		-18,4
F(2,26) = 1,690; p = 0,2041	Int (*10^2)	7,67	0,6	6,50	0,6	5,9	0,3		-7,2		-19,0		-12,7
F(2,27) = 2.114; p = 0,1403	C.int.	2,6	0,7	2,0	0,4	1,2	0,1		-25,1		-52,7		-36,9
**Cg1** (3,36; 3,2; 3,04)													
F(2,26) = 0,6083; p = 0,5518	Density	15,8	2,7	14,4	1,8	17,6	1,8		-8,8		11,0		21,7
F(2,25) = 1,419; p = 0,2608	Int (*10^2)	9,6	1,0	8,5	1,1	8,7	0,9		-11,0		-9,7		1,4
F(2,25) = 1,791; p = 0,1875	C.int.	8,3	1,6	7,2	1,6	8,6	1,0		-13,0		4,0		19,5
**IL** (3,36;3,2; 3,04)													
F(2,24) = 9.73; p = 0,0008***	Density	**19,7**	**2,2**	**32,1**	**1,9**	**22,8**	**1,9**	0,0007***	62,7	0,3655	15,4	0,0135*	-29,0
F(2,25) = 3,904; p = 0,0335*	Int (*10^2)	**13,4**	**2,2**	**7,9**	**0,8**	10,1	0,8	0,0027*	-41,0	0,2173	-24,8	0,5089	27,4
F(2,25) = 0,1120; p = 0,8945	C.int.	14,8	2,9	13,2	2,3	13,7	1,7	0,6793	-10,5	0,7623	-6,9	0,8524	4,0
**PrL** (3,36; 3,2; 3,04)													
F(2,26) = 0,8800; p = 0,4268	Density	21,3	3,7	17,7	2,8	16,2	1,6	0,6405	-16,8		-24,1		-8,8
F(2,26) = 0,9459; p = 0,4013	Int (*10^2)	9,8	0,8	8,7	0,7	10,1	0,8	0,5611	-11,7		2,5		16,2
F(2,26) = 0,6719; p = 0,5194	C.int.	12,9	3,2	9,1	2,2	9,8	1,7		-29,1		-24,0		7,2
**LO** (3,36; 3,2; 3,04)													
F(2,26) = 3.859; p = 0,0341*	Density	**41,8**	**4,4**	**27,4**	**3,9**	40,2	3,9	0,0493*	-34,5	0,7963	-3,7	0,07	47,1
F(2,25) = 1.088; p = 0,3523	Int (*10^2)	6,1	0,8	4,8	0,2	5,4	0,6	0,3254	-21,5	0,8285	-11,7		12,5
F(2,25) = 3,332 p = 0,0522	C.int.	12,0	1,2	7,7	0,9	11,9	1,9		-35,6	0,9951	-0, 83	0,0714	35,2
**VO** (3,36; 3,2; 3,04)													
F(2,26) = 3,606; p = 0,0415*	Density	**60,4**	**6,3**	43,6	5,1	45,8	2,1	0,0500*	-27,8	0,0949	-24,3	0,9450	4,8
F(2,25) = 0,01244; p = 0,9876	Int (*10^2)	4,6	0,4	4,6	0,3	4,7	0,5	>0,999	0,2	0,9896	1,8	0,9904	1,6
F(2,25) = 1,283; p = 0,2948	C.int.	15,1	2,1	11,1	1,7	12,3	1,5	0,2753	-26,3	0,5093	-18,8	0,8821	10,2
**Pir** (3,36; 3,2; 3,04)													
F(2,25) = 0,2273; p = 0,7983	Density	28,5	2,1	26,7	3,6	25,2	3,7	0,9269	-6,3	0,7804	-11,5	0,9449	-5,5
F(2,25) = 1,640; p = 0,2141	Int (*10^2)	11,7	1,0	9,1	0,9	11,1	1,2	0,2296	-22,0	0,9194	-5,1	0,3687	21,7
F(2,25) = 0,3960; p = 0,6722	C.int.	19,1	2,1	15,0	3,5	15,6	3,3	0,6543	-21,5	0,8388	-18,2	0,9391	4,2
**PVA** (-1,8; -1,96; -2,12)													
F(2,27) = 0,8299; p = 0,4469	Density	51,8	7,5	40,8	4,4	50,5	7,5		-21,3		-2,4		24,0
F(2,26) = 1,186; p = 0,3210	Int (*10^2)	9,7	1,0	9,1	1,0	7,8	0,8		-7,1		-20,2		-14,2
F(2,26) = 1,706; p = 0,2013	C.int.	17,4	2,6	11,6	1,4	14,4	2,3		-33,3		-17,4		23,9
**VLGMC** (-4,14; -4,3; -4,46)													
F(2,27) = 5,166; p = 0,0252*	Density	**40,3**	**1,5**	33,9	1,9	**33,3**	**2,1**	0,0314*	-15,9	0,0209*	-17,4	0,9689	-1,8
F(2,27) = 2.169; p = 0,1338	Int (*10^2)	15,9	0,9	13,6	0,8	14,2	0,8		-14,8		-10,6		5,0
F(2,26) = 6.482; p = 0,005**	C.int.	**23,1**	**1,8**	**16,6**	**1,1**	**17,3**	**1,2**	0,0078**	-27,9	0,0168*	-25,2	0,9418	3,8
**CA3V** (-4,3; -4,46; -4,62)													
F(2,26) = 1,467; p = 0,2490	Density	14,1	3,0	9,6	1,6	14,2	1,9		-32,2		0,4		48,1
F(2,26) = 2,733; p = 0,837	Int (*10^2)	10,5	1,1	9,7	0,8	7,8	0,5		-7,9		-25,4		-19,0
F(2,26) = 1,700; p = 0,2023	C.int.	8,0	1,5	5,2	1,0	6,1	0,7		-34,8		-24,4		16,0
**DG** (-4,3; -4,46; -4,62)													
F(2,23) = 0,8389; p = 0,4450	Density	19,1	0,7	15,6	2,0	16,3	1,8		-18,5		-14,8		4,6
F(2,23) = 1,400; p = 0,2668	Int (*10^2)	12,4	1,4	13,0	0,7	11,1	0,7		4,4		-10,6		-14,4
F(2,23) = 1,099; p = 0,3501	C.int.	12,8	1,9	11,6	1,4	9,8	1,0		-9,2		-23,4		-15,6

Analyzed brain areas are listed from the most rostral position to the most caudal position; in bold mean values and p-values of the density, as number of cells per area,the intensity or cumulative intensity significantly different from control or from the other CMS group. Int.: intensity; C.int: cumulative intensity. A vs C, anhedonic versus control; R vs C, resilient group versus control; R vs A, Resilient versus anhedonic group. %diff., difference among groups, in percentage; D+M: dark and medium stained *c-Fos* expressing cells One-way ANOVA. p < 0.05;

** p < 0.01;

*** p < 0.001;

data is presented as group means (±SEM).

### Densitometry

**Fig 4:**
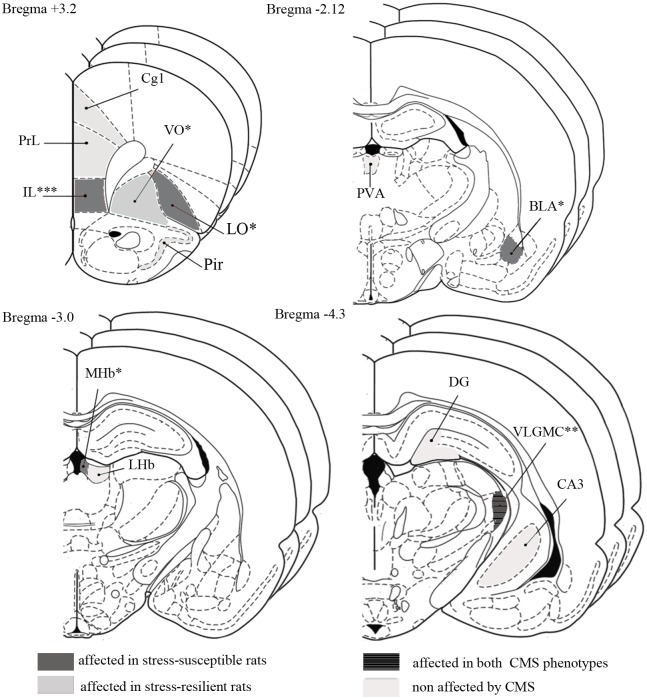
Figure modified from the BRAIN ATLAS [31]. Pictures show 13 different substructures analysed at 3 rostrocaudal levels, within each structure, throughout different brain regions. In grey areas affected in stress-susceptible rats; in dark grey with stripes brain regions affected in both CMS phenotypes; in light grey areas affected in stress resilient rats. In beige, areas not affected by the CMS. DG, dendate gyrus; Cg1, cingulate cortex—area 1; IL, infralimbic cortex; PrL, prelimbic cortex; LO, lateral orbital cortex; VO, ventral orbital cortex; Pir, piriform cortex; PVA, paraventricular thalamic nucleus—anterior part; BLA, basolateral amygdaloid nucleus—anterior part; MHb, medial habenula; LHb, lateral habenula; CA3, field CA3 of hippocampus; VLGMC, ventral lateral geniculate nucleus magnocellular part; * p < 0.05; ** p < 0.01; *** p < 0.001.

We analysed c-Fos-ir within 13 different brain areas. These areas include the anterior part of basolateral daloid nucleus (BLA), MHb and LHb, area 1 of the cingulate cortex (Cg1), the prelimbic (PrL) and infralimbic (IL) cortex, the lateral orbital (LO) and ventral orbital (VO) cortex, piriform cortex (Pir), the anterior part of the paraventricular thalamic nucleus (PVA), the magnocellular part of the ventral lateral geniculate nucleus (VLGMC), the CA3 of the ventral hippocampus (CA3V), and the dentate gyrus (DG). An overview of the 13 brain areas with the anterior-posterior localization of sections included for the analysis is shown in Fig 4; mean ± SEM of c-Fos-ir intensity, cumulative intensity and density is shown in Table 1.

**Fig 5:**
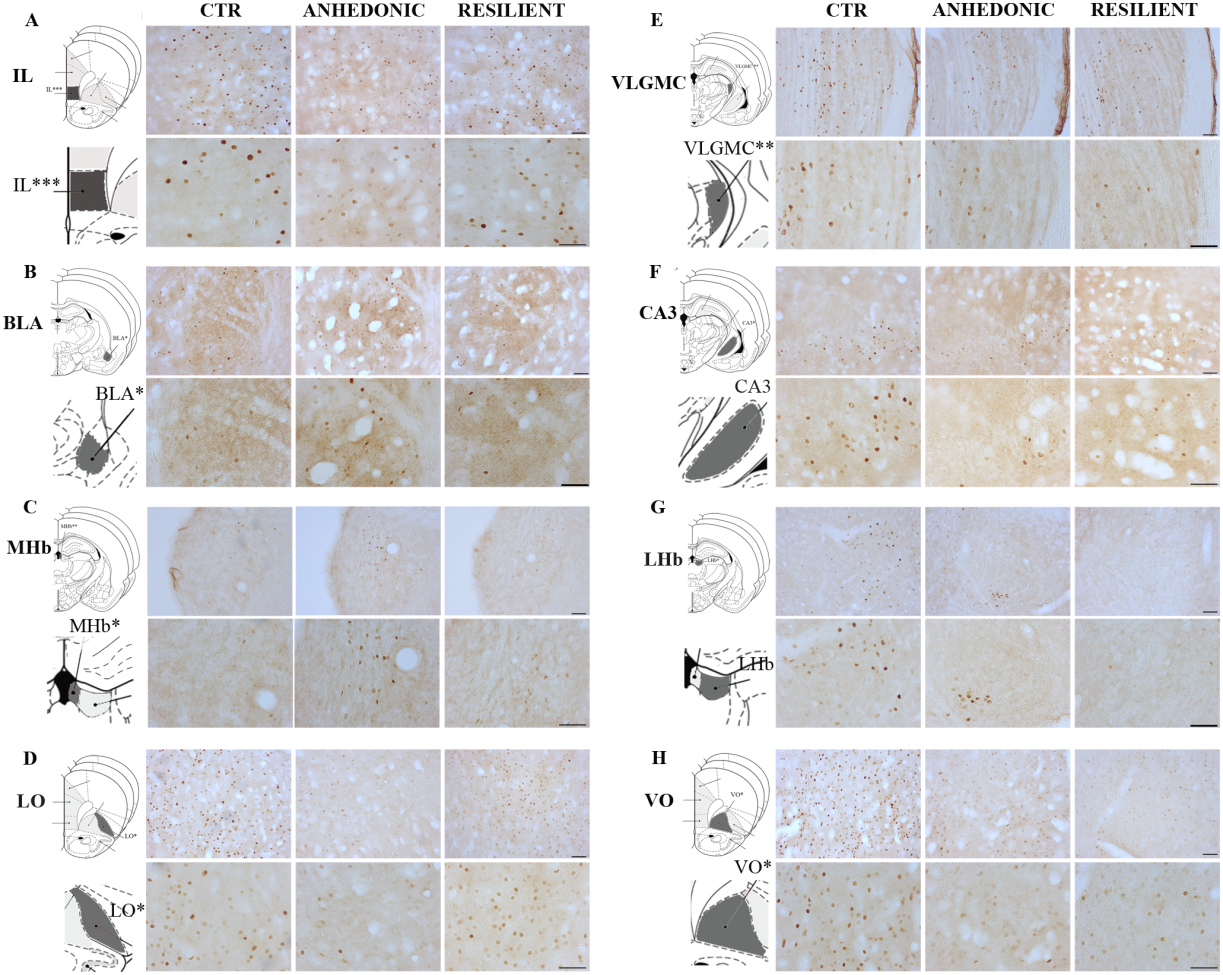
Representative microphotograph of c-Fos expression in different brain areas. High and low magnification pictures of representative sections to compare c-Fos-ir in eight brain regions. IL, infralimbic cortex; LO, lateral orbital cortex; VO, ventral orbital cortex; BLA, basolateral amygdaloid nucleus—anterior part; MHb, medial habenula; LHb, lateral habenula; CA3, field CA3 of hippocampus; VLGMC, ventral lateral geniculate nucleus; magnocellular part. Each panel shows a high and low magnification picture of the area, and a picture of the corresponding area modified from the rat brain atlas [31]. Scale bar in panel A-H = 50um.

Significant changes of c-Fos-ir was found in respectively the BLA, MHb, IL, VLGMC, VO, LO, and a tendency towards changes in the CA3V (p = 0.078). Hence, CMS exposure did not induce any significant changes of c-Fos-ir within the Cg1, PrL, Pir, LHb, PVA, and DG (Fig 5A–5H and Table 1).

The c-Fos-ir intensity within the BLA of resilient animals was significantly decreased relative to the anhedonic-like animals (25%, F (2,26) = 3,779); p = 0.00363, Fig 5B and Table 1), however, the c-Fos-ir density of the BLA was comparable within the two groups.

In the anhedonic-like animals the c-Fos-ir intensity within the MHb was significantly increased relative to the control animals (26%, F (2,27) = 4,331; p = 0.0327), while c-Fos-ir densities were comparable between the two groups (Fig 5C and Table 1).

Although in the LHb the c-Fos-ir in the resilient group was lower when compared to the control group (19%), this was insignificant (F (2,26) = 1,690, p = 0.2; Fig 5G and Table 1).

From analysis of the prefrontal cortex (both PrL and IL) we found only significant changes in the IL. Exposure to CMS reduced c-Fos-ir intensity within the IL of anhedonic-like animals when compared to the control group (41%, F (2,25) = 3,904; p = 0.0027). Meanwhile the reversed effect was observed upon c-Fos-ir cell density measures of the IL, since the c-Fos-ir density of anhedonic-like animals was increased relative to the control animals (63%, F (2,24) = 9,73; p = 0.0007). Conversely a reduction of c-Fos-ir density was found when the resilient animals were compared to the anhedonic-like animals (29%, p = 0.0135) (Fig 5A, Table 1).

Within LO, the c-Fos-ir density of the anhedonic-like animals was significantly reduced when compared to the control animals (35%, F (2,26) = 3,859; p = 0.0493). In this area of the brain there was also a tendency towards a reduced c-Fos-ir associated with anhedonic animals compared to the resilient animals (47%, p = 0.07). This tendency was associated with a decrease in the cumulative density (47%; p = 0.07) within the LO in the resilient animals versus the anhedonic rats (54% F (2,25) = 3,332; p = 0.07(Fig 5D and Table 1).

Despite the lack of c-Fos-ir differences between anhedonic-like and resilient animals within the VO, c-Fos-ir density within the VO of the anhedonic animals was significantly decreased compared to the control animals (27,8%, F (2,26 = 3,859); p = 0.0500) (Fig 5H, Table 1).

The analysis revealed that the c-Fos-ir densities in the VLGMC of both the anhedonic-like animals (16%, F (2,27) = 5,166; p = 0.0314) and resilient animals (17%, p = 0.0209) were reduced significantly when compared to the control animals (Fig 5E and Table 1). This effect was also reflected by the cumulative intensities (anhedonic-like compared to control, 28%, F (2,26) = 6,482; p = 0.0078; resilient compared to control, 25%, p = 0.0168).

Analysis of the CA3 revealed a tendency towards a reduction in c-Fos-ir intensity of the resilient animals when compared to the control group (25%, p = 0.078), although this was insignificant (Fig 5F and Table 1).

## Discussion

The ability of stressful life events to induce depression varies substantially among individuals suggesting an intrinsic genetic vulnerability in humans, as well as in laboratory animals [34] [35]. CMS is a validated animal model of stress-induced anhedonia [30, 36, 37] [38, 39] [40], in which the unpredictable sequential presentation of microstressors appropriately mimic human life stressors. In the present study we showed a general effect of CMS exposure on sucrose consumptions, the anhedonic rats decreased sucrose intake more than 30%, while resilient rats decreased the sucrose intake less than 10%. Our results demonstrate that the CMS model identifies genetically vulnerable animals to stress, similar to human cases, which is in line with previously reported data [30].

In the present study we were able to show the stress effect both at the behaviour level as measured by sucrose intake, but also at the level of neuronal activity in specific brain regions, by measuring *c-Fos* expression, a marker of neuronal activity [41]. We used quantitative densitometry in a comprehensive analysis of *c-Fos* expression within 13 different brain areas in stress susceptible and stress resilient rats, respectively. Our results show that CMS induced drastic changes in *c-Fos* expression in several brain areas such as the limbic structures (like the MHb, and the BLA), the prefrontal cortex, the lateral and ventral orbital cortex and the VLGMC, while no differences were detected in areas mainly related to memory and learning such as the dentate gyrus, the cingulate cortex the ventral hippocampus or the piriform cortex. Previous research has shown that chronic stress decreases *c-Fos* mRNA in some areas of the brain including the paraventrivular nucleus of the hypothalamus (PVN), the medial prefrontal cortex and the basolateral amygdala, while other areas such as piriform cortex, amygdala, hippocampus, thalamus and lateral habenula were unaffected [42]. High and low anxiety behavioural traits have also been shown to associate with changes in *c-Fos* expression in some areas of the brain involved in fear/anxiety circuitries. Both low and high anxiety behavioural rat lines showed a decrease in *c-Fos* expression in the cingulate cortex as well as an increase in various cortical, limbic and hypothalamic areas [19]. However in most of the previous studies, the changes in *c-Fos* expression were demonstrated either by mRNA quantification or by measuring the density (number of cells per mm^2^) of immunoreactive c-Fos cells in distinct areas rather than using a quantification method to measure the intensity level [42] [19].

Previous studies also reported a differential cellular staining for c-FOS varying from very intense (dark brown) to very light (light brown), with the intensity level being proportional to the expression level, when stained with diaminobenzidine. The distribution in the brain was region specific with moderate/dark staining in areas that were involved in stress responses such as the limbic structure and in those involved in neuroendocrine circuits [43].

In the present study we firstly applied stereology to investigate whether c-Fos-ir would confirm an impact of stress exposure upon neuronal activation within the LHb and MHb, as this has been shown to be the case in previous studies on acute stress [44]. Stereology is a very exact and unbiased technique for quantification of cells [45], however measurements of staining parameters such as cumulative cell area and staining intensity of all c-Fos-ir cells are both complicated and still insufficient using standard stereology software and techniques. Stereological estimates did not reveal any changes in c-Fos-ir cell number in the LHb and MHb. After microscopic analysis of brain sections, we found that the differences in *c-Fos* expression were not only restricted to the number of cells expressing *c-Fos*, but also to the intensity of the staining in each individual c-Fos-ir cell.

Therefore, to have measurements on staining/expression intensity in individual cells we further analysed these regions using densitometry. Densitometry is a biased quantification method and is restricted to densities and numbers and thus based upon a variety of assumptions.

Our data on cell density, using densitometry and stereology, revealed that densitometry provided a measure of the cell density comparable to the stereological quantification of total numbers. However, despite no differences in c-Fos-ir cell numbers or density was found, the densitometry analysis revealed significant differences of expression intensity in the MHb.

In the habenula subregions we have shown that although stress did not increase the number of c-Fos-ir cells in a specific region, measured as density, stress did affect *c-Fos* expression measured as the intensity of the c-Fos-ir cells in the MHb.

Our study showed that c-Fos-ir intensity was lower in the resilient group when compared to the control in the LHb, although this was not significant. We did not find any significant difference of c-FOS level in the LHb, in the anhedonic group. This can be explained by the fact that the LHb is activated during acute stress so it is possible that we would have seen a more pronounced response in the anhedonic group in the acute phase of the CMS paradigm.

We also need to take into consideration that the maximal level of the c-FOS is detectable after 1.3 hours, after an acute event, and gradually decrease 4–6 hours after acute exposure [46–48]. However, increased *c-Fos* mRNA is detectable up to one month following the cessation of chronic variable stress [42].

The habenula is a key structure mediating the response to stressful situations and emotionally negative stimuli [49, 50]. It has been demonstrated that the habenula is hyperactive during depression [51]. Our findings confirmed the previous studies of stress effects upon the habenular complex [44] [52, 53].

Our data also showed a significant increase in c-Fos-ir intensity in the MHb in the anhedonic group, when compared to the control group. The increase of c-Fos-ir intensity in the MHb is also in line with the involvement of this area in modulation of the immune response during stress [54].

To detect the difference in *c-Fos* expression, we quantified the c-Fos-ir intensity of all individual c-Fos-ir profiles in all the selected areas using densitometry. We also included the measurement of cumulative c-Fos-ir intensity in the analysis, which takes into account the combined effect of the intensity and the number/density of cells.

The anatomical network that is underlying emotional behaviour has been well established from brain lesion studies in animals and further confirmed recently by new neuroimaging technologies, suggesting a strong network between the amygdala, the medial prefrontal cortex, and the orbital cortex and an involvement of these areas in mood disorders [55] [56] [57]. Based on this we analyzed cFos-ir within 13 different brain areas, which are all listed in Table 1.

Our results showed a significant decrease of the c-Fos-ir intensity in the resilient group compared to the anhedonic, despite of a similar density for the two groups in the amygdala. Although no significant changes were detected in the cingulate cortex in the present study, we showed a dramatic decrease of the c-Fos-ir intensity in the IL in the anhedonic group compared to the resilient and control groups. Interestingly, the reduction of c-Fos-ir intensity in the anhedonic group was associated to a drastic increase of c-Fos-ir density when compared to the control animals, indicating once again the relevance of the changes in *c-Fos* expression rather than the number of c-Fos positive neurons. Given the recognized link between the amygdala and prefrontal cortex, our data support the link between these two areas and their susceptibility during stressful conditions [58].

Our data showed that chronic mild stress induced *c-Fos* increased expression only in some areas of the brain, while decrease has been detected in other areas such as in the infralimbic region, where a reduction of more than 40% of c-Fos-ir intensity compared to the control group was detected in the anhedonic group, despite a similar number of cell density. This dual susceptibility of brain areas to stress has been reported previously. Rat lines bred for high anxiety-related behaviour (HAB) showed an increase in *c-Fos* expression in several areas of the brain including hypothalamus, habenula, and a decrease in other areas such as in the prelimic, infralimbic, cingulate cortex, when compared to rat lines bred for low anxiety-related behaviour (LAB), suggesting that specific areas of the brain are reactive and associating with anxiety [19, 58, 59].

Our study showed that although a similar number of cells, in a region of interest like amygdala, express the c-Fos protein, the expression level was lower in the resilient animals.

The amygdala, as well as the hippocampus, is an area involved in processing anxiety related emotions and plays a key role in reappraisal of a specific emotional valens to a sensory input [60, 61], however much less is known about the circuits that are activated during stressful conditions per se [62, 63].

Our data showed no changes in c-Fos-ir intensity in anhedonic group compared to the control in the CA3. However, the analysis of c-Fos expression in the CA3 region revealed a decrease of c-Fos-ir in the resilient animals compared to the control, in line with previous studies that show a decrease of c-Fos in the hippocampus as a consequence of the adaptive response to stress [64].

The investigation of VO and LO, which are areas involved in emotional and social behaviour and learning, showed a significant reduction of 35% in the c-Fos-ir number of cells expressing *c-Fos* per area in the anhedonic group, when compared to the control group in the LO (p = 0.04), which is also associated with a tendency of reduction of the cumulative intensity in the anhedonic group when compared to the resilient group in the cells expressing *c-Fos* (p = 0.07). Conversely, the density of cells expressing *c-Fos* in the resilient group was increased with more than 40% when compared to the anhedonic animals. In the VO we detected a decrease of the of *c-Fos* density in the resilient group compared to the control.

Interestingly, during examination of the brain sections with low-power lenses we observed obvious differences of c-Fos-ir within individual animals in the VLGMC. VLGMC is not an area normally associated with stress and depression, however in order to address the observations quantitatively we decided to include the VLGMC in our densitometry analysis. Our data showed that stress induce a significant decrease in the density of the c-Fos-ir positive cells, accompanied by a significant decrease in the cumulative intensity in both resilient and anhedonic groups, when compared to the control group. As recently shown in a mice study, light induces *c-Fos* activation in some brain areas such as LHb, but not in others such as DLG (dorsal lateral geniculate), where light rather cause a decrease of *c-Fos* activation [65]. It was reported that light does not induce changes in *c-Fos* in retinorecipient areas such as in the LHb, dorsal lateral geniculate (DGL), in the ventral subparalventricular zone (vSPZ), suprachiasmic nucleus (SCN) or in the VLGMC of night active grass rats [65, 66]. Our data are in accordance with other previous studies showing that CMS animals display circadian rhythm disturbances [67] which are common symptoms of MDD [68]. These can provide a possible association between CMS exposure and the decrease of *c-Fos* expression in the VLGMC area. The intermittent illumination that the rats have to undergo as part of the stressors in the CMS model, might explain the changes detected in the VLGMC area that commonly is affected by light.

Electrophysiological studies using rats showed that not only neurons in the VLGMC, but also neurons in the habenula, responded to light and the *in vitro* recording showed a circadian rhythm in the firing rate [69]. Although little is known about the neural mechanism responsible for these differences, the present work highlights the activation of *c-Fos* in specific brain regions in the CMS model.

We found that chronic mild stress led to a prolonged specific alteration of selected brain areas involved in limbic network and this might have a crucial role in mediating the activities and the information processing in the brain. Furthermore our analysis of the LHb and MHb showed that there was an effect on c-Fos-ir intensity upon stress in the MHb, despite no changes in the cell number/profile density in these areas using either stereology or densitometry. This finding demonstrates the importance of considering c-Fos-ir intensity as a marker of neuronal activation rather than the cellular number.

## Supporting information

S1 Fig Figure representing the method for imageJ analysis. Panel A the original figure, in B black and white image with the detected *c-Fos* expressing cells detected by the program and marked in yellow. Panel C the mask created by the program. Panel D and E show low and high magnification of two proximal cells, respectively, correctly recognized and measured as two distinct cells. Scale bar in panel A = 150um.
(TIF)

## Abbreviations

BLA: basolateral amygdaloid nucleus—anterior part
CA3: field CA3 of hippocampus
Cg1: cingulate cortex—area 1
DG: dendate gyrus
IL: infralimbic cortex
LHb: lateral habenula
LO: lateral orbital cortex
MHb: medial habenula
Pir: piriform cortex
PrL: prelimbic cortex
PVA: paraventricular thalamic nucleus—anterior part
VLGMC: ventral lateral geniculate nucleus magnocellular part
VO: ventral orbital cortex
