# Supplementary figures and images for: Neuronal substrates underlying stress resilience and susceptibility in rats
Source: PLoS One. 2017 Jun 16;12(6):e0179434. doi: 10.1371/journal.pone.0179434 (PMC5473563; doi:10.1371/journal.pone.0179434)

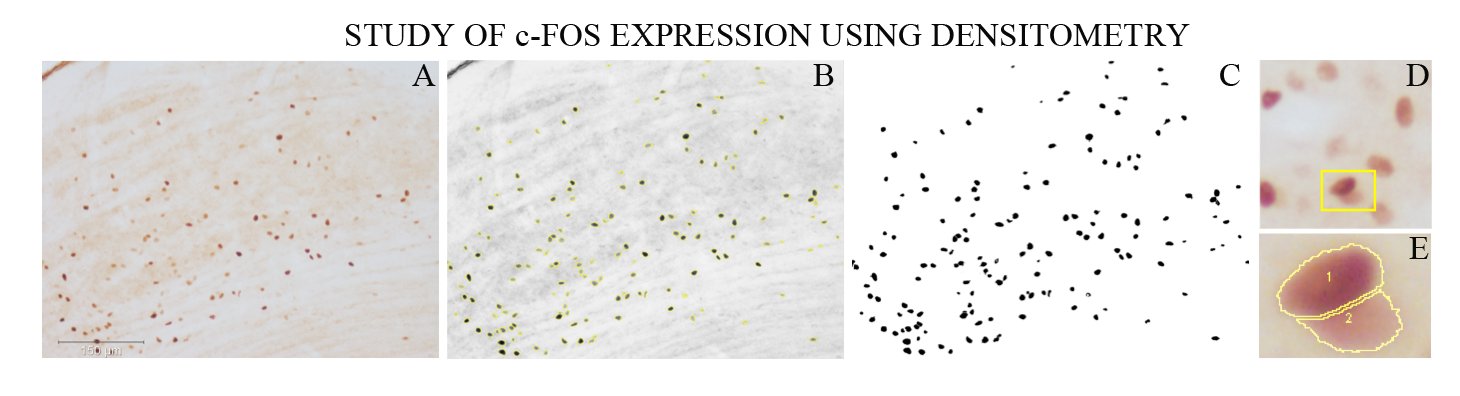

Supplement: S1 Fig — Panel A the original figure, in B black and white image with the detected c-Fos expressing cells detected by the program and marked in yellow. Panel C the mask created by the program. Panel D and E show low and high magnification of two proximal cells, respectively, correctly recognized and measured as two distinct cells. Scale bar in panel A = 150um. (TIF) [file pone.0179434.s001.tif]
